# Multiple breath washout testing in adults with pulmonary disease and healthy controls – can fewer measurements eventually be more?

**DOI:** 10.1186/s12890-017-0543-y

**Published:** 2017-12-11

**Authors:** Frederik Trinkmann, Johannes Götzmann, Daniel Saur, Michele Schroeter, Katharina Roth, Ksenija Stach, Martin Borggrefe, Joachim Saur, Ibrahim Akin, Julia D. Michels

**Affiliations:** 1 1st Department of Medicine (Cardiology, Angiology, Pulmonary and Intensive Care), University Medical Centre Mannheim, Medical Faculty Mannheim, Heidelberg University, Theodor-Kutzer-Ufer 1-3, 68167 Mannheim, Germany; 2 DZHK (German Centre for Cardiovascular Research), partner site Mannheim, University Medical Centre Mannheim, Medical Faculty Mannheim, Heidelberg University, Theodor-Kutzer-Ufer 1-3, 68167 Mannheim, Germany

**Keywords:** Multiple breath washout, Lung clearance index, Duplicate measurements, Pulmonary disease, Adults

## Abstract

**Background:**

Multiple breath washout (MBW) became a valuable research tool assessing ventilation heterogeneity. However, routine clinical application still faces several challenges. Deriving MBW parameters from three technically acceptable measurements according to current recommendations prolongs test times. We therefore aimed to evaluate reporting only duplicate measurements in healthy adults and pulmonary disease.

**Methods:**

One hundred and fifty-three subjects prospectively underwent conventional lung function testing and closed-circuit SF_6_-MBW. Three technically acceptable MBW-measurements were obtained in 103 subjects.

**Results:**

Lung clearance index (LCI) differed significantly among 19 controls (7.4 ± 0.8), 19 patients with sarcoidosis (8.1 ± 1.2), 32 with bronchial asthma (9.2 ± 1.9) and 33 with COPD (10.8 ± 2.2, *p* < 0.001). Within-test repeatability was high (coefficient of variation between 2.5% in controls and 3.6% in COPD) and remained unchanged when only including the first two measurements. Likewise, LCI remained stable with mean absolute changes ranging from 0.9 ± 0.8% in controls to 1.5 ± 0.9% in COPD (*p* = 0.1). Mean test time reduction differed significantly between groups reaching 200 s in COPD (*p* = 0.01).

**Conclusions:**

Duplicate SF_6_-MBW-measurements are sufficient in adult patients with pulmonary disease and healthy controls. LCI values and intra-test repeatability are not affected reducing total test time statistically significant. Our findings have the potential to further facilitate application of MBW in research and clinical routine.

**Trial registration:**

NCT03176745, June 2, 2017 retrospectively registered.

**Electronic supplementary material:**

The online version of this article (10.1186/s12890-017-0543-y) contains supplementary material, which is available to authorized users.

## Background

Multiple breath washout (MBW) testing has been first described in the 1950s [1, 2] for the assessment of ventilation heterogeneity. Clearance of a tracer gas from the lungs is measured most commonly using exogenous sulphur hexafluoride (SF_6_) or endogenous nitrogen (N_2_). In recent years, MBW has become a valuable research tool and numerous studies were performed focusing on paediatric populations and patients with cystic fibrosis or bronchiectasis [3–5]. Little data is available for adults although promising results for diagnosis and prediction of chronic obstructive pulmonary disease (COPD) became available recently [6–8]. Nevertheless, several challenges exist and still prevent MBW from routine clinical application. Open wash-in SF_6_-MBW using a mass spectrometer is considered to be the gold standard [9] while being associated with high costs and effort and not holding regulatory approval. As a consequence, indirect techniques such as N_2_-MBW have been experiencing a renaissance. Pure oxygen is used for N_2_ wash-out while directly measuring oxygen and carbon dioxide concentrations. Considerable inaccuracies can therefore be introduced due to measurement errors in respiratory gas concentrations, N_2_ back diffusion and changes in breathing pattern caused by 100% oxygen [10, 11]. In contrast, SF_6_ concentrations can be directly measured with high accuracy using a newly developed photo-magneto-acoustic multi-gas analyser [12, 13]. This allowed the construction of a portable device for SF_6_-MBW [14] with considerably lower gas concentrations as compared to mass spectrometry using 4% SF_6_ [11, 15] and is associated with significant cost reduction while environmental effects and maintenance become negligible. Moreover, the system has regulatory approval by the U.S. Food and Drug Administration and CE marking allowing its use in clinical routine.

Recently, publication of an ERS/ATS consensus statement was an important step to standardization of the technique in general [9]. Reporting MBW parameters from three technically acceptable measurements is recommended independently of the test gas used. However, this can lead to prolonged duration in N_2_-MBW where wash-out is time critical while in SF_6_-MBW wash-in and costs are relevant factors. For both tracer gases, several adaptions to the measurement protocols have been proposed including reduction of total measurements or earlier cut-offs for terminating wash-out [16–19]. Moreover, a closed circuit setup for SF_6_-MBW was shown to effectively reduce wash-in times further facilitating application [20]. Therefore, the aims of our study were to prospectively evaluate (1) the feasibility of closed-circuit SF_6_-MBW in healthy adults and in pulmonary disease, (2) the influence of reporting duplicate measurements on lung clearance index (LCI), with-in test repeatability and total test time.

## Methods

### Subjects

The total collective consisted of 153 subjects including pulmonary healthy controls (*n* = 24) as well as patients suffering from COPD (*n* = 50), bronchial asthma (*n* = 54) and sarcoidosis (*n* = 25). All participants were in clinically stable condition and written informed consent was obtained prior to inclusion. The study protocol was approved by our local ethics committee and registered at clinicaltrials.gov (NCT03176745). Controls had normal lung function testing including whole-body plethysmography and transfer factor, no previously diagnosed pulmonary disease as well as no respiratory symptoms. Lung function testing including the shapes of flow-volume and flow-pressure curves was independently assessed by two experienced investigators. Detailed information on classification criteria of controls and pulmonary disease is given in the Additional file 1. Patients in unstable clinical condition, with infective lung disease or need for long term oxygen therapy were not included.

### Study protocol

All subjects underwent three consecutive MBW tests in upright position followed by whole-body plethysmography (MasterScreen Body, CareFusion 234 GmbH, Höchberg, Germany). Functional residual capacity (FRC) was determined from end-expiratory shutter manoeuvres during normal breathing. If obstruction was present (FEV_1_ / vital capacity < 80% of predicted), we performed reversibility testing in patients with COPD (88%), bronchial asthma (66%), or sarcoidosis (37%), respectively. Doses of 40 μg ipratropium bromide and 100 μg fenoterol hydrobromide were administered using a soft-mist haler (Berodual Respimat, Boehringer Ingelheim Pharma GmbH & Co. KG, Germany). Transfer factor (TLCO) was determined in single breath technique in all subjects.

### Multiple breath washout

For MBW testing, we used a commercially available device (Innocor, PulmoTrace ApS, Glamsbjerg, Denmark). The closed-circuit system consists of a 3-l rebreathing bag filled with a mixture of room air and test gas (94% O_2_, 1% SF_6_ and 5% N_2_O, PulmoTrace ApS) from an on-board gas cylinder as previously described in detail [20]. FRC and LCI were derived from three consecutive wash-outs using proprietary software provided by the manufacturer (software version 8.0 beta 1). Subjects were breathing tidally and the test was stopped when end tidal SF_6_ had fallen to less than 1/40th of the starting concentration. Only patients with three technically acceptable measurements based on slightly modified ATS/ERS criteria were included in final analysis (Additional file 1).

### Statistical analysis

Data was analysed using MedCalc version 17.4 (MedCalc Software, Mariakerke, Belgium). Mean values are given ±SD unless stated otherwise. Differences between disease entities were assessed by ANOVA for continuous variables or Chi-squared test for categorical variables. Student’s t-test was used to evaluate differences between patients with successful and unsuccessful measurements as well as duplicate and triplicate measurements, respectively. For the duplicate measurement approach, mean LCI values were derived from the first two runs. The coefficient of variation (CV) was calculated as SD/mean from duplicate and triplicate MBW measurements. Mean percentage changes in LCI are given as absolute values (modulus) to facilitate comparison with CV. A planned subgroup sample size of 20 would provide 80% power for detecting a difference of 1 ± 1.5% in LCI. An alpha error of less than 5% in two-sided testing was considered statistically significant.

## Results

A total of 103 subjects were included in the final analysis. Baseline characteristics and lung function data are given in Table 1. Mean LCI differed significantly between controls (7.4 ± 0.8), sarcoidosis (8.1 ± 1.2), bronchial asthma (9.2 ± 1.9) and COPD (10.8 ± 2.2, *p* < 0.001, ANOVA). Within-test repeatability was high with an overall CV of 3.1%, ranging from 2.5% in controls to 3.6% in COPD (Table 2). FRC as determined by plethysmography yielded significantly higher values as compared to MBW in patients with COPD (3.8 ± 0.9 vs. 3.0 ± 0.8 p < 0.001) and asthma (2.9 ± 0.8 vs. 2.4 ± 0.7, *p* < 0.05). In contrast, no differences were found in controls (3.2 ± 0.5 vs. 3.0 ± 0.6, *p* = 0.1) or sarcoidosis (3.1 ± 0.7 vs. 2.9 ± 0.7, *p* = 0.4).

**Table 1:** Baseline characteristics and lung function data (analysed collective, *n* = 103)

		Control (*n* = 19)		COPD (*n* = 33)		Asthma (*n* = 32)		Sarcoidosis (*n* = 19)		
	Unit	Mean ± SD	Range	Mean ± SD	Range	Mean ± SD	Range	Mean ± SD	Range	*p*-value^#^
Age	years	56 ± 22	21 – 85	66 ± 10	46 – 86	59 ± 16	27 – 88	51 ± 10	37 – 79	<0.01
Height	cm	172 ± 10	157 – 198	168 ± 6	157 – 182	166 ± 10	145 – 188	174 ± 8	150 – 187	<0.05
Weight	kg	80 ± 21	50 – 132	81 ± 14	50 – 120	80 ± 17	45 – 117	76 ± 19	47 – 128	0.8
BMI	kg/m^2^	27.1 ± 7.6	17.5 – 49.1	28.8 ± 4.9	20.3 – 42.5	28.8 ± 6.2	16.1 – 44.5	25.6 ± 7.2	13.4 – 46.5	0.3
Smoker										
Never	n (%)	15 (79)		1 (3)		14 (44)		12 (63)		<0.001
Current	n (%)	2 (11)		8 (24)		6 (19)		1 (5)		
Former	n (%)	2 (11)		24 (73)		12 (38)		6 (32)		
*RT*	n (%)	–		29 (88)		21 (66)		7 (37)		0.8
Positive	n (%)	–		8 (28)		6 (29)		1 (14)		
FEV_1_/VC	% pred	98 ± 8	84 – 114	73 ± 17	42 – 103	89 ± 11	58 – 110	92 ± 11	70 – 110	<0.001
FEV_1_	% pred	96 ± 14	65 – 127	65 ± 20	36 – 106	86 ± 18	38 – 110	89 ± 16	57 – 112	<0.001
TLC	% pred	103 ± 10	87 – 116	110 ± 19	72 – 148	105 ± 17	65 – 141	100 ± 15	83 – 139	0.2
VC	% pred	96 ± 16	64 – 119	91 ± 23	41 – 142	98 ± 21	59 – 136	96 ± 16	67 – 135	0.5
RV	% pred	122 ± 21	82 – 177	147 ± 33	108 – 211	123 ± 19	81 – 156	112 ± 22	69 – 158	<0.001
RV/TLC	% pred	113 ± 17	86 – 152	129 ± 19	100 – 184	115 ± 14	91 – 151	105 ± 13	75 – 123	<0.001
TLCO/VA	% pred	99 ± 9	88 – 114	63 ± 18	28 – 95	83 ± 13	62 – 112	85 ± 16	44 – 122	<0.001
FRC_pleth_	l	3.2 ± 0.5	2.4 – 4.0	3.8 ± 0.9	2.1 – 5.8	2.9 ± 0.8	1.7 – 4.8	3.1 ± 0.7	1.6 – 4.2	<0.001

*BMI* body mass index, *RT* reversibility testing, *FEV*_*1*_ forced expiratory volume in 1 s, *VC* vital capacity, *TLC* total lung capacity, *RV* residual volume, *TLCO* transfer factor for carbon monoxide corrected for alveolar volume, *FRC*_*pleth*_ functional residual capacity in plethysmography, *% pred* percent of predicted, *SD* standard deviation

^#^ *p*-values between groups were calculated using ANOVA for continuous variables or Chi-squared test for categorical variables, respectively

**Table 2:** Multiple breath washout data (*n* = 103)

		Triplicate measurements			Duplicate measurements			*p*-value^#^	
		Mean ± SD	Range	CV ± SD	Mean ± SD	Range	CV ± SD	Means	CVs
Control									
LCI		7.4 ± 0.8	6.0 – 8.6	2.5 ± 1.8	7.4 ± 0.8	5.9 – 8.6	2.7 ± 2.1	0.6	0.6
FRC_MBW_	l	3.0 ± 0.6	1.9 – 4.2	3.6 ± 3.0	2.9 ± 0.6	1.8 – 4.1	4.1 ± 3.9	0.5	0.3
Test time	s	469 ± 134	211 – 709		314 ± 85	141 – 467		<0.0001	
COPD									
LCI		10.8 ± 2.2	8.0 – 17.6	3.6 ± 1.8	10.9 ± 2.2	7.8 – 17.0	3.1 ± 2.3	0.7	0.1
FRC_MBW_	l	3.0 ± 0.8	1.5 – 4.8	4.0 ± 4.0	2.9 ± 0.8	1.5 – 4.8	2.7 ± 3.3	0.1	<0.05
Test time	s	592 ± 182	294 – 1165		392 ± 122	191 – 775		<0.0001	
Asthma									
LCI		9.2 ± 1.9	5.9 – 13.6	3.3 ± 1.7	9.2 ± 1.9	5.9 – 13.9	3.1 ± 2.4	0.1	0.4
FRC_MBW_	l	2.4 ± 0.7	1.0 – 4.1	3.9 ± 3.9	2.4 ± 0.7	1.0 – 4.2	4.1 ± 4.8	0.2	0.7
Test time	s	462 ± 153	242 – 1022		307 ± 101	172 – 690		<0.0001	
Sarcoidosis									
LCI		8.1 ± 1.2	5.8 – 11.3	2.7 ± 1.7	8.2 ± 1.2	5.8 – 11.1	2.2 ± 2.3	0.4	0.1
FRC_MBW_	l	2.9 ± 0.7	1.5 – 4.0	3.8 ± 3.3	2.9 ± 0.7	1.5 – 4.1	3.4 ± 3.4	0.3	0.5
Test time	s	500 ± 174	228 – 970		328 ± 156	156 – 622		<0.0001	
Overall									
LCI		9.2 ± 2.2	5.8 – 17.6	3.1 ± 1.8	9.2 ± 2.2	5.8 – 17.0	2.9 ± 2.3	0.2	<0.05
FRC_MBW_	l	2.8 ± 0.8	1.0 – 4.8	3.9 ± 3.6	2.8 ± 0.8	1.0 – 4.8	3.5 ± 3.9	<0.05	0.2
Test time	s	510 ± 171	211 – 1165		338 ± 112	141 – 775		<0.0001	

*LCI* lung clearance index, *FRC*_*MBW*_ functional residual capacity in multiple breath washout, *SD* standard deviation, *CV* coefficient of variation [%], *CI* 95%-confidence interval

^#^ *p*-values between means of measurement groups were calculated using paired Student’s t-test

**Fig. 1:**
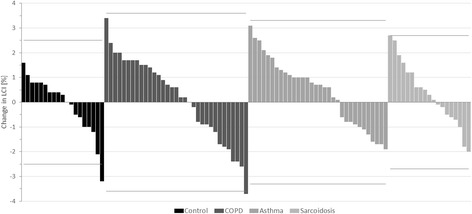
Change in lung clearance index (LCI). Waterfall diagram of the LCI percentage change when comparing duplicate and triplicate measurements (*n* = 103). No differences in mean absolute changes (modulus) were found between controls (0.9 ± 0.8%), COPD (1.5 ± 0.9%), sarcoidosis (1.1 ± 0.8%) and asthma (1.3 ± 0.7%) in ANOVA (*p* = 0.1). Solid grey lines indicate mean coefficient of variation (CV) of the respective group with only two datasets marginally exceeding within-test repeatability

**Fig. 2:**
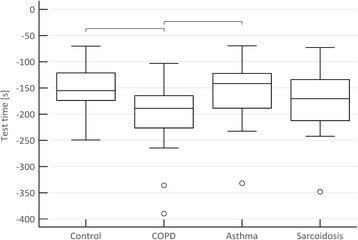
Test time reduction. Absolute test time reductions for duplicate as compared to triplicate measurements (*n* = 103). Means ranging from 155 ± 49 s in controls to 200 ± 62 s in COPD. Connectors indicate statistically significant differences (*p* = 0.01, ANOVA)

When only including the first two MBW measurements, LCI remained stable in all groups with mean absolute changes (modulus) of 0.9 ± 0.8% in controls, 1.5 ± 0.9% in COPD, 1.1 ± 0.8% in sarcoidosis and 1.3 ± 0.7% in asthma, respectively (p = 0.1, ANOVA, Fig. 1). Within-test repeatability was not negatively affected when only including two instead of three MBW measurements. Overall CV significantly decreased from 3.1 to 2.9% (p < 0.05, t-test). Total test times differed significantly in all groups when comparing the duplicate to triplicate measurement approach (Table 2). Mean test time reductions ranged from 155 s in controls to 200 s in COPD (*p* = 0.01, ANOVA, Fig. 2) lying within the variation of an individual test in the respective group.

**Fig. 3:**
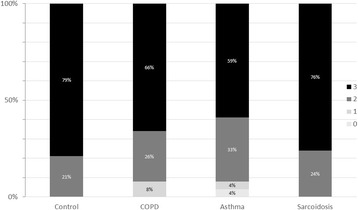
Successful measurements. No difference in the number of successful measurements was found between controls, COPD, asthma, or sarcoidosis (*p* = 0.3, Chi-squared test) in the overall collective (*n* = 153)

We had to exclude datasets from 50 subjects representing 33% of the screened collective due to at least one invalid measurement. A minimum of two technically acceptable MBW measurements could be obtained in 92% of COPD, in 93% of bronchial asthma, in all sarcoidosis patients as well as in all healthy controls from the complete cohort (Fig. 3). There was no difference in the number of successful measurements between groups (*p* = 0.3, Chi-squared test). In patients with at least two successful measurements (*n* = 145), an average 84% of valid trials were obtained from the first two out of three (Additional file 1: Figure S1). To further assess factors associated with an unsuccessful MBW test, we analysed baseline and lung function data of the complete collective screened. Subjects with unsuccessful measurements had significantly higher TLC (112 ± 19 vs. 105 ± 16% of predicted) and RV (142 ± 43 vs. 129 ± 28% of predicted, *p* < 0.05 each, t-test) as compared to subjects with complete data sets. Moreover, excluded patients were more frequently current smokers (50 vs. 17%, *p* < 0.05, Chi-squared test) with details given in (Additional file 1: Table S1).

## Discussion

We were able to demonstrate that determination of LCI derived from SF_6_-MBW is feasible in adults with pulmonary disease and healthy controls. Values are not altered when only reporting two instead of three technically acceptable measurements. Mean changes were considerably lower than within-test repeatability in all groups and overall test times could be noticeably reduced. LCI differed among all groups and yielded the highest readings in COPD.

Until now, data for comparison of diseases other than cystic fibrosis or bronchiectasis is scarce for our SF_6_-based setup. The feasibility of N_2_-MBW in patients with COPD was recently evaluated by Fähndrich and co-workers. An increasing ventilation heterogeneity in patients with airway obstruction or hyperinflation was found with a mean LCI of 12.6 as compared to 7.0 in healthy controls [8]. However, it has been demonstrated that N_2_-MBW reproducibly yields higher absolute LCI readings than SF_6_-MBW [21, 22]. Much of this difference can be potentially explained by differing physiological properties and the aforementioned technical aspects associated with the test gases [11, 21, 23]. N_2_ has a higher diffusion rate and smaller molar mass as compared to SF_6_ resulting in a more proximal diffusion-convection front [9]. Moreover, SF_6_ may not reach very poorly ventilated regions during wash-in while endogenous N_2_ may prolong wash-out from these regions resulting in higher LCI values. N_2_ back diffusion plays an important role becoming most pronounced at the end of the wash-out where it may contribute 20% of the measured signal [23] and increases with longer wash-out times [24]. Recent findings convincingly support this explanation as N_2_-excretion was demonstrated to increase with cardiac output under exercise conditions simultaneously measuring both tracer gases [10]. Previously, excretion rates were found to fit a multi-phase exponential curve although varying intra- and inter-individually. Application of correction equations has shown to significantly reduce the effect of tissue N_2_, however, it cannot be eliminated completely. While also not affecting the interpretation of treatment effects, application of tissue N_2_ correction equations is therefore currently not recommended [24]. As a consequence, values acquired with either technique should not be used interchangeably complicating direct comparison of studies using different tracer gases.

In controls, we found good agreement for FRC as determined by body plethysmography and MBW, respectively. This is in accordance with previous in-vitro validation studies using the open-circuit approach [12, 13]. Only ventilated areas will contribute to FRC determination using MBW whereas compressible gas volumes are measured in body plethysmography [22]. This relates to significant differences in FRC between the two techniques seen in patients with obstructive ventilation disorders in our collective. In general, deriving FRC from panting manoeuvres yields accurate results in controls as well as milder obstructive disease when controlling panting frequency [25, 26]. With increasing disease severity, body plethysmography was shown to systematically overestimate lung volume as compared to both computed tomography and Helium dilution [27]. However, there is no consent about the ideal technique for measuring lung volumes. Our measurement setup allows determination of FRC from end-expiratory shutter manoeuvres during normal breathing. This overcomes important impediments to the panting-based approach such as incomplete equilibration of mouth and alveolar pressures and does not lead to additional hyperinflation or increase in end-expiratory pressures. Computed tomography may be affected by postural lung volume changes [28] and incomplete inspiration while Helium dilution may result in underestimation due to gas trapping [29]. Interestingly, gas trapping independently predicts patients with a larger difference of plethysmography and computed tomography derived total lung capacity [30] and inter-modal differences even were postulated as a diagnostic tool differentiating COPD severity [31].

Within-test repeatability found in our study is comparable to previously published data in stable adults showing CVs between 3.2 and 4.5% using SF_6_ as tracer gas [4, 20, 32]. Closed-circuit tracer gas wash-in has been demonstrated to reduce wash-in times by 32 to 50% in cystic fibrosis patients and healthy controls [20] as compared to the conventional open-circuit technique. We could achieve another 34% reduction of total test time when only reporting two technically acceptable measurements. Therefore, a complete dataset allowing calculation of LCI can be acquired in less than 7 min on the average even in patients with COPD using SF_6_. In contrast, N_2_ based measurements are usually more time consuming and wash-out times increase with disease severity in cystic fibrosis [33]. In patients with severe COPD, durations of up to 20 min have been reported for a single measurement leading to a low rate of successful measurements of 55% [8]. Although mean test time reductions due to omitting a third measurement should not be overemphasized in our collective, time savings can add up to over 6 min in an individual patient. Mean overall rejection rate was 33% which is comparable to a previous report by Jensen where 27% of N_2_-MBW measurements were excluded after a standardized review process [34]. In our collective, patients suffering from obstructive ventilation disorders such as COPD and bronchial asthma showed the highest rates of 34 and 41%, respectively. Patients with unsuccessful measurements had higher TLC and RV values corresponding to hyperinflation. In both obstructive disorders, the majority of valid tests were obtained from the first two runs. Notably, in the trial by Jensen up to seven measurements were performed until the operator determined that three good trials were obtained or the subject was unable to continue testing. In contrast, we did not repeat invalid tests in our protocol calculating success rates from a set of three consecutive MBW measurements.

When applying adult quality control criteria to a paediatric population, within-test repeatability could be significantly reduced from 8.5 to 4.7% using SF_6_. However, success rates were as low as 41% beyond infancy and could be increased to an overall 70% using preschool recommendations [17]. In our collective, two valid measurements were obtained from the first two runs in an average 4 out of 5 patients. The smallest benefit of an additional third measurement was seen in patients with COPD where 96% of successful trials were acquired from the first two runs. In context of the lower overall success rates in patients with airway obstruction, this is an important finding and reducing time and effort needed is crucial for clinical application of MBW. Moreover, it has been hypothesized that inclusion of poorly ventilated lung regions not reached during the initial but subsequent trials could potentially increase LCI values. This effect should become more pronounced in severe obstructive disease [8]. Percentage changes were distributed quite homogenously in our collective including increases as well as decreases when comparing duplicate versus triplicate LCI measurements. This is in accordance with previous findings where LCI and FRC values remained unchanged in patients with cystic fibrosis [17].

When interpreting our results, several points should be taken into consideration. Although we included a broad spectrum of pulmonary disorders, interstitial lung disease is underrepresented being restricted to patients with sarcoidosis. While lung disease affecting lung parenchyma may not necessarily increase ventilation heterogeneity, MBW testing could be potentially beneficial in disease entities affecting peripheral airway function. These may include respiratory bronchiolitis–associated interstitial lung disease (RB-ILD) or cryptogenic organizing pneumonia (COP). Our investigation focuses on LCI as the predominant MBW parameter reported in the literature. Therefore, our findings should not be uncritically transferred to other indices such as phase III analyses or moment ratios. Considerably larger CVs have been shown for parameters of conductive (S_cond_) and acinar (S_acin_) ventilation heterogeneity [7]. At the same time, lower success rates are postulated as a result of the more elaborate underlying algorithm. Due to the cross-sectional design, we are not able to provide estimates of the minimal clinical important difference for SF_6_-MBW. From technical considerations, it should be possible to detect small longitudinal changes due to the high intra-test repeatability while further research is required. Nevertheless, our findings add important information to the scarce data available on adult MBW testing. For the first time, we could demonstrate the feasibility of SF_6_-MBW in a variety of pulmonary disorders in a large adult collective. Moreover, we included a wide range of LCI values and age classes meeting the requirements of both research and clinical applications.

## Conclusions

We were able to demonstrate that duplicate LCI measurements derived from SF_6_-MBW are sufficient in adult patients with COPD, bronchial asthma, sarcoidosis as well as in healthy controls. LCI values and intra-test repeatability are not affected while total test time is reduced statistically significant. Our findings have the potential to further facilitate application of MBW testing in research and daily clinical routine.

## Additional file

Additional file 1: Classification criteria. Acceptability criteria for MBW. **Table S1.** Analysis of excluded subjects. **Figure S1.** Success rates for the first two out of three trials in patients with at least two successful measurements. (DOCX 194 kb)

## Abbreviations

COP: Cryptogenic organizing pneumonia
COPD: Chronic obstructive pulmonary disease
CV: Coefficient of variation
FRC: Functional residual capacity
LCI: Lung clearance index
MBW: Multiple breath washout
N_2_: Nitrogen
RB-ILD: Respiratory bronchiolitis–associated interstitial lung disease
RV: Residual volume
S_acin_: Acinar ventilation heterogeneity
S_cond_: Conductive ventilation heterogeneity
SF_6_: Sulphur hexafluoride
TLC: Total lung capacity
TLCO: Transfer factor
