# Supplementary material for: Multiple breath washout testing in adults with pulmonary disease and healthy controls – can fewer measurements eventually be more?
Source: BMC Pulm Med. 2017 Dec 11;17:185. doi: 10.1186/s12890-017-0543-y (PMC5725793; doi:10.1186/s12890-017-0543-y)
Supplement: Additional file 1: — Classification criteria. Acceptability criteria for MBW. Table S1. Analysis of excluded subjects. Figure S1. Success rates for the first two out of three trials in patients with at least two successful measurements. (DOCX 194 kb) [file 12890_2017_543_MOESM1_ESM.docx]

**Additional File**

**Classification criteria**

- **Healthy controls**
  - no history of pulmonary disease
  - absence of dyspnoea, cough, thoracic pain
  - self-reported smoking history < 10 pack years
  - normal lung function testing
    - normal shape of flow-volume curve
    - normal shape of flow-pressure curve
    - FEV_1_/VC >80%, TLC > 80%, TLCO/VA > 80% (all % of predicted)
- **COPD**
  - clinical history and/or specialist diagnosis of COPD
  - risk factor(s) (tobacco smoking, α_1_-antitrypsin-deficiency, biomass fuel exposure)
  - persistent bronchial obstruction (FEV_1_/VC <80% of predicted) and/or hyperinflation (RV/TLC >40%, clubbing in flow-pressure curve) and/or radiological sign of emphysema, each without alternative explanation
  - dyspnoea, cough and/or sputum production
- **Bronchial asthma**
  - clinical history and/or specialist diagnosis of bronchial asthma
  - respiratory symptoms compatible with asthma varying over time (wheeze, dyspnoea, chest tightness, cough)
  - variable and/or reversible obstructive ventilation disorder and/or airway hyperresponsiveness
  - exclusion of alternative explanation
- **Sarcoidosis**
  - clinical history and/or specialist diagnosis of sarcoidosis
  - lymphocytic alveolitis and CD4/CD8 > 3.5 in bronchoalveolar lavage and/or noncaseating epithelioid granuloma
  - exclusion of alternative explanation (particularly tuberculosis and lymphoma)

**Acceptability criteria for MBW**

- breathing pattern
  - stable tidal volume, no hyperventilation, no hypoventilation
  - no coughing
  - assessed clinically and in-silico
- variability
  - within 10% from the median FRC / LCI across triplicate tests
  - careful examination for technical issues if larger, but within 25%
  - rejection if larger > 25%
- no leak
  - equilibration between inspiratory and expiratory SF_6_ concentrations during wash-in
  - no sudden drop in inspiratory SF_6_ concentration during wash-in
- test termination
  - at least three consecutive breaths with end tidal SF_6_ concentrations <1/40th of starting value
- test conduction
  - no excessive swallowing (clinical assessment)
  - sufficient interval between tests

(twice the wash-out time or measurement of resituated gas concentrations)

| Table S1 Analysis of excluded subjects | | | | | | |
| --- | --- | --- | --- | --- | --- | --- |
|  |  | Successful (n=103) | | Unsuccessful (n=50) | |  |
|  |  | Mean ± SD | Range | Mean ± SD | Range | p-value^#^ |
| Age | years | 59 ± 15 | 21 - 88 | 56 ± 19 | 21 - 89 | 0.2 |
| Height | cm | 169 ± 9 | 145 - 198 | 169 ± 9 | 146 - 186 | 0.6 |
| Weight | kg | 80 ± 17 | 45 - 132 | 78 ± 20 | 50 - 125 | 0.6 |
| BMI | kg/m^2^ | 27.9 ± 6.4 | 13.4 - 49.1 | 27.3 ± 6.0 | 18.4 - 42.9 | 0.6 |
| *Smoker* |  |  |  |  |  |  |
| never | n (%) | 42 (41) |  | 34 (41) |  | <0.05 |
| current | n (%) | 17 (17) |  | 17 (50) |  |  |
| former | n (%) | 44 (43) |  | 16 (27) |  |  |
| *RT* | n (%) | 57 (55) |  | 35 (70) |  | >0.9 |
| positive | n (%) | 15 (26) |  | 9 (26) |  |  |
| FEV_1_/VC | % pred | 86 ± 16 | 42 - 114 | 82 ± 18 | 35 - 115 | 0.2 |
| FEV_1_ | % pred | 81 ± 21 | 36 - 127 | 83 ± 28 | 27 - 143 | 0.7 |
| TLC | % pred | 105 ± 16 | 65 - 148 | 112 ± 19 | 68 - 151 | <0.05 |
| VC | % pred | 95 ± 20 | 41 - 142 | 100 ± 23 | 41 - 156 | 0.2 |
| RV | % pred | 129 ± 28 | 69 - 211 | 142 ± 43 | 65 - 292 | <0.05 |
| RV/TLC | % pred | 117 ± 18 | 75 - 184 | 123 ± 25 | 58 - 182 | 0.2 |
| TLCO/VA | % pred | 80 ± 19 | 28 - 122 | 75 ± 23 | 24 - 122 | 0.2 |
| FRC_pleth_ | l | 3.3 ± 0.8 | 1.7 - 5.8 | 3.4 ± 1.1 | 1.4 - 7.1 | 0.4 |
| BMI: body mass index, RT: reversibility testing, FEV_1_: forced expiratory volume in 1 sec, VC: vital capacity, TLC: total lung capacity, RV: residual volume, TLCO: transfer factor for carbon monoxide corrected for alveolar volume, FRC_pleth_: functional residual capacity in plethysmography, % pred: percent of predicted, SD: standard deviation. ^#^p-values between groups were calculated using unpaired Student’s t-test for continuous variables or Chi-squared test for categorical variables, respectively. | | | | | | |

**Figure S1**

**
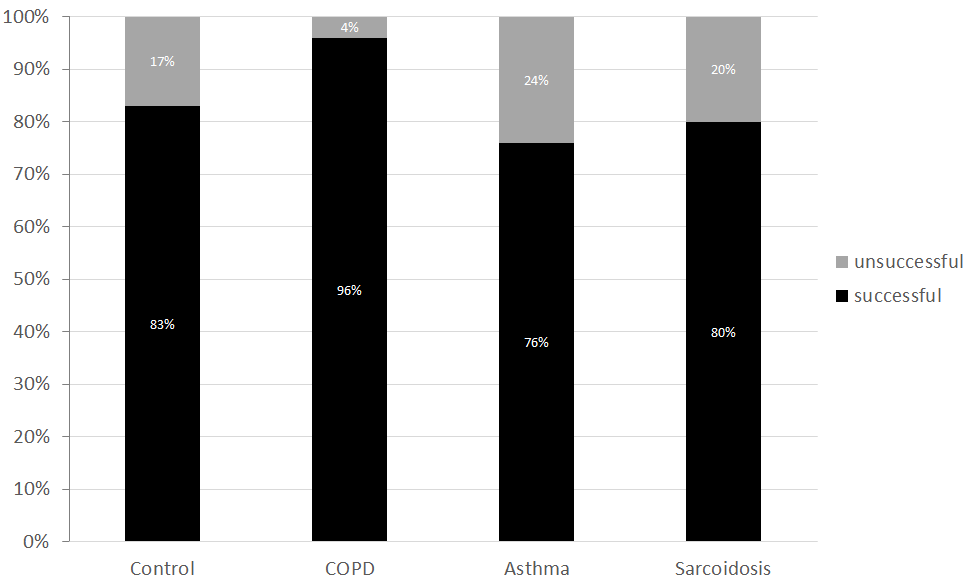
**

Success rates for the first two out of three trials in patients with at least two successful measurements (n=145) showing an average of 84% (p<0.1, Chi-squared test).
